# Association of angiotensin-converting enzyme insertion/deletion (ACE I/D) gene polymorphism with susceptibility to prostate cancer: an updated meta-analysis

**DOI:** 10.1186/s12957-022-02812-x

**Published:** 2022-11-04

**Authors:** Jianhui Du, Jianhua Lan, Hai Yang, Qiao Ying, Guohua Huang, Jian Mou, Jia Long, Zhenghua Qiao, Qiyi Hu

**Affiliations:** 1 Department of Urology, People’s Hospital of Guang’an City, Guang’an, 638000 Sichuan China; 2 Department of Urology, People’s Hospital of Guang’an District, Sichuan 638000 Guang’an, China; 3 Department of Urology, Affiliated Longhua People’s Hospital, Southern Medical University (Longhua People’s Hospital), No. 38, Jinglongjianshe Road, Longhua, Shenzhen, 518109 Guangdong China

**Keywords:** Angiotensin-converting enzyme (ACE), Susceptibility, Prostate cancer, Meta-analysis

## Abstract

**Objective:**

This meta-analysis aims to explore the association between angiotensin-converting enzyme (ACE) insertion/deletion (I/D) gene polymorphism and susceptibility to prostate cancer (PCa).

**Methods:**

We searched studies related to ACE I/D polymorphism and susceptibility to PCa through PubMed, Web of Science, Embase, Cochrane Library, and Scopus databases from inception to June 1, 2022. Five gene models, including allelic, dominant, recessive, homozygote, and heterozygote models, were analyzed. The pooled odds ratio (OR) was calculated using Stata 15.0 software. Publication bias was judged by the funnel plot and Egger’s test, with the robustness of the findings verified by sensitivity analysis.

**Results:**

Eight published articles (including ten studies) were identified. The pooled results showed that ACE I/D locus polymorphism was significantly correlated with the risk of PCa under all gene models except for the heterozygous model (D vs. I: OR= 1.58, 95% CI: 1.14–2.21; DD vs. DI+II: OR=1.68, 95% CI: 1.11–2.54; DD+DI vs. II: OR=1.76, 95% CI: 1.11–2.80; DI vs. II: OR= 1.44, 95% CI: 0.99–2.10; DD vs. II: OR= 2.12, 95% CI: 1.15–3.93). Subgroup analysis based on genotype frequencies in the control group meeting Hardy-Weinberg equilibrium showed statistically significant differences in all gene models. The funnel plot and Egger’s test indicated no publication bias. The sensitivity analysis verified the robustness of the conclusions obtained in this meta-analysis.

**Conclusion:**

ACE I/D locus polymorphism correlates to PCa risk. Allele D, genotype DD+DI, and DD at the ACE I/D locus increase susceptibility to PCa and can therefore serve as a potential diagnostic and screening molecular marker for PCa patients.

**Supplementary Information:**

The online version contains supplementary material available at 10.1186/s12957-022-02812-x.

## Introduction

The incidence and mortality of cancer are increasing year by year globally, and in 2018, cancer has become the leading cause of death, resulting in more than 9.6 million deaths worldwide [1]. According to the statistics in 2019, prostate cancer (PCa) was a male malignant tumor with the widest distribution and highest prevalence in developed countries, and its incidence had surpassed that of lung cancer and ranks first among males [2]. This latest analysis also predicted that in 2020 in the USA, 191,930 new males would suffer from PCa, accounting for 21% of all new cancer cases and showing an increasing trend compared to less than 20% before 2018; about 33,330 diagnosed patients eventually die for it, with the mortality rate second only to lung cancer among men [2]. In 2020, PCa in men over 50 years of age resulted in 3.5 million years of life loss, with 40% of patients occurring in men aged over 75 [3]. This cancer imposes a huge global burden and is expected to increase over time [3]. Therefore, identifying risk factors for PCa is essential to obtain an insight into his disease and contributes to exploring possible therapeutic measures.

Angiotensin-converting enzyme (ACE), also known as kininase II, is a crucial position in the renin-angiotensin-aldosterone system (RAAS) system [4]. ACE is a zinc-containing peptidyl dipeptidase consisting of 1306 amino acids [4]. ACE has homologous domains, and is widely expressed in humans, such as in coronary arteries, renal vascular endothelial cells, and capillary endothelial cells [5–7]. The ACE gene contains 26 exons and 25 introns, with a length of approximately 21 kb and a location at chromosome 17q23 [8]. The ACE gene has a 287-bp insertion/deletion (I/D) polymorphism in intron 16 and is divided into homozygous II (490 bp), homozygous DD (190 bp), and heterozygous ID [8].

A meta-analysis by Wang et al. [9] in 2018 determined a correlation between ACE I/D polymorphism and PCa susceptibility, but this study had significant limitations of a small number of included articles and inadequate robustness of the conclusion. Afterward, there are still many individual studies published on ACE I/D polymorphism and PCa susceptibility [10–12]. Therefore, this study used meta-analysis to investigate the association between the two in order to provide evidence-based medical evidence for proving whether ACE I/D polymorphism can be considered as a potential diagnostic and screening molecular biomarker in PCa patients.

## Methods

This study was performed under the guidelines of the Preferred Reporting Items for Systematic Reviews and Meta-analyses (PRISMA) [13]. Online registration has been accepted by PROSPERO (CRD42022356555).

### Literature retrieval

Relevant studies on ACE I/D polymorphism and susceptibility to PCa were collected from PubMed, Web of Science, Embase, Cochrane Library, and Scopus databases, with time span from inception to June 1, 2022. The language was limited to English. The main combined search terms were as follows: (“Angiotensin-converting enzyme” OR “ACE”) AND (“Prostate cancer” OR “prostate carcinoma” OR “prostate tumor”) AND (“polymorphism” OR “single nucleotide polymorphism” OR “mutation”). The detailed search results in Pubmed were provided in Supplementary Table 1. Additionally, the references of included qualified studies were further tracked to obtain more favorable qualified literature. Literature retrieval was done by two authors jointly, and their results were cross-checked. Besides, the discussion was required in cases of disagreements.

### Inclusion and exclusion criteria

Articles satisfying all the following criteria were included (1) literature providing evaluation on the relationship between ACE I/D polymorphism and PCa risk directly and indirectly; (2) a case-control or cohort study; (3) with PCa patients as study subjects; (4) with non-PCa cases (healthy population or hospital controls) as controls; and (5) English literature. Exclusive criteria were as follows: (1) cell experiments, animal experiments, letters, conference abstracts, and reviews; (2) literature without valid raw data; (3) duplicate reports of the same batch of specimens; and (4) the Newcastle-Ottawa Scale (NOS) score less than six.

### Literature quality assessment and data extraction

The NOS scale [14] assessed the risk of bias of the included studies based on three categories: selection of cases and controls, comparability between cases and controls, and exposure/outcome. The total score of NOS is nine points, and our analysis was restricted to high-quality studies, as defined by a NOS score equal to or higher than six. Two authors independently screened articles, and then extracted the data from eligible ones and evaluated their quality. Their results were cross-checked, and discussion was required if disagreed. The extracted data mainly included first author, publication time, region, age, source of the control group, genotyping technique, and the number of each genotype of the cases and controls.

### Statistical analysis

Data processing was performed using Stata 15.0 (Stata Corporation, College Station, TX, USA). Five gene models were analyzed, which were the allelic model (D vs. I), dominant model (DD+DI vs. II), recessive model (DD vs. DI+II), homozygote model (DD vs. II), and heterozygote model (DI vs. II). The odds ratio (OR) and 95% confidence interval (CI) were used to assess the strength of the association between ACE I/D polymorphism and susceptibility to PCa. Furthermore, the heterogeneity among studies was assessed by Cochrane’s *Q* test and *I*^2^ statistic [15]. if *I*^2^ ≥ 50% or *P* ≤ 0.1, indicating significant heterogeneity, the data were combined using the random-effects model (REM); conversely, the fixed-effects model (FEM) was used. Subgroup analyses based on ethnicity and Hardy-Weinberg equilibrium (HWE) were carried out to investigate the source of heterogeneity. Potential publication bias was measured using the funnel plot and Egger’s regression test. Finally, the robustness of the conclusions obtained was verified by sensitivity analysis. The specific operation of sensitivity analysis was to conduct a meta-analysis again after eliminating each study and then compared the differences between the results of the re-combination and those before exclusion. *P* < 0.05 was considered statistically significant.

## Results

### Search results and basic characteristics of included studies

**Fig. 1:**
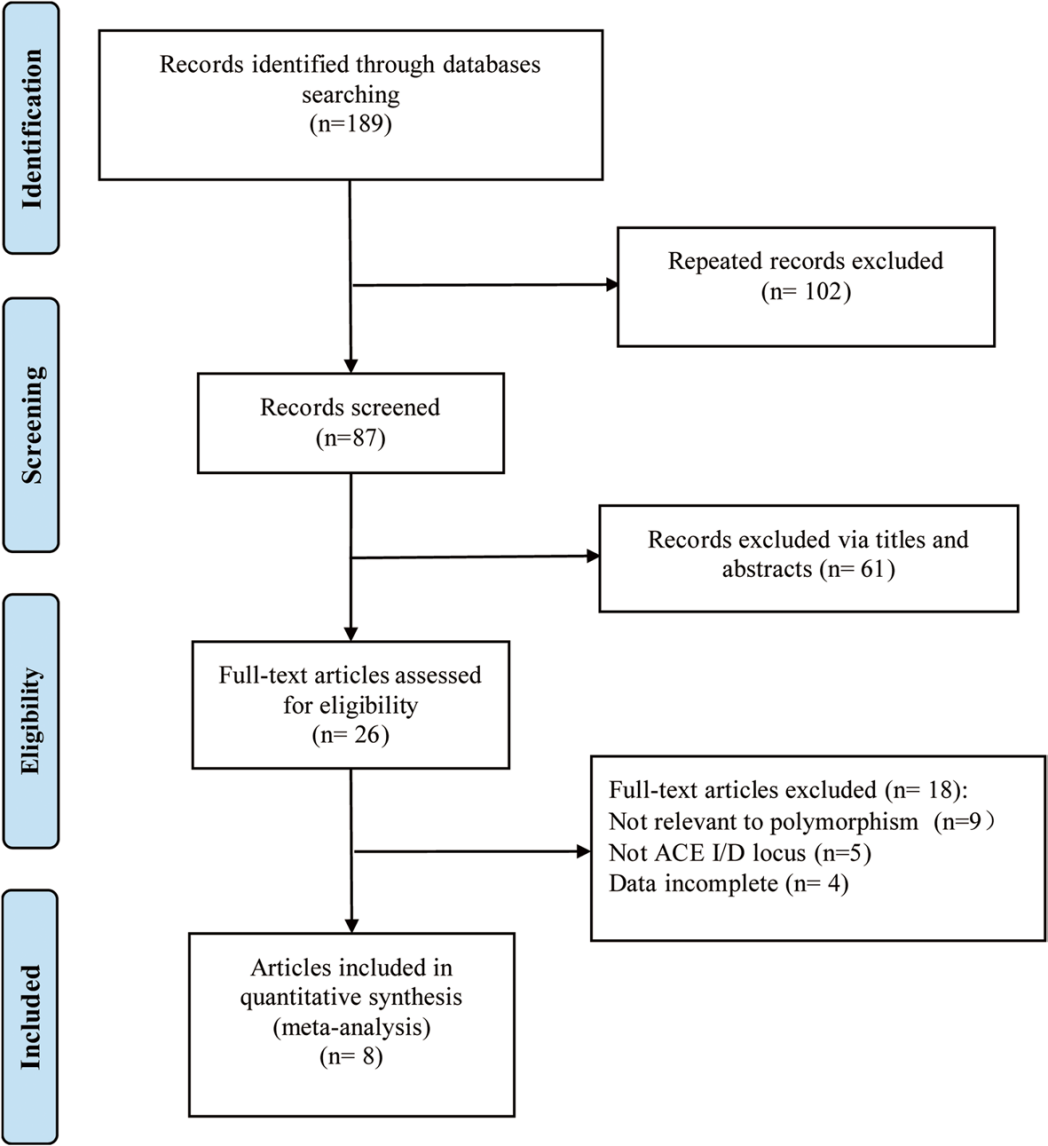
Screening flow chart of included literature

A total of 189 articles were retrieved, and 26 were obtained after excluding duplicated ones and reading titles and abstracts (Fig. 1). Next, the full text of the remaining articles was reviewed. Finally, eight eligible literature was identified [10–12, 16–20], including 817 patients with PCa and 6917 controls. Two of the eight [10, 11] reported two different controls, so the two articles contained four studies. Finally, 10 studies were included in this meta-analysis, with eight studies in Caucasian populations, one in the Chinese population, and one in the Mexican population with basic characteristics in Table 1, with all included studies high-quality studies having NOS scores greater than six points.

**Table 1:** Basic characteristics of the included studies

Study	Year	Region	Con source	Number	Ages (cases/con)	Genotyping method	Cases			Con			HWE	NOS score
				Cases/con			II	DI	DD	II	DI	DD		
Sierra [18]	2009	Mexico	PB	19/28	NR	PCR-RFLP	0	7	12	9	12	7	0.464	8
van der Knaap [17]	2008	Netherlands	PB	209/6015	70.4±9.7/70.4±9.7	PCR	44	109	56	1332	3006	1677	0.828	8
Wang [19]	2012	China	HB	189/290	71.58±7.69/72.51±8.10	PCR	49	84	56	115	135	40	0.970	7
Yigit [16]	2007	Turkey	PB	48/51	70.55±9.68/69.02±7.89	PCR-RFLP	4	19	25	12	24	15	0.692	8
Hasanzad [20]	2013	Iran	HB	84/96	67±8.7/67±8.7	PCR-RFLP	18	27	39	16	32	48	0.014	8
Said [12]	2021	Tunisia	PB and HB	124/143	70.78 ± 9.258 /69.67 ± 9.03	PCR	10	50	64	27	58	58	0.075	8
Shubbar a [10]	2018	Iraq	PB	75/81	68.03±5.73/56.42±3.95	PCR	10	20	45	36	18	27	0.000	8
Shubbar b [10]	2018	Iraq	HB	75/75	68.03±5.73/49.28±6.10	PCR	10	20	45	20	35	20	0.564	8
Asmahan a [11]	2020	Lebanon	PB	69/69	66.4±8.5/55.8±11.0	PCR	7	43	19	8	34	27	0.582	8
Asmahan b [11]	2020	Lebanon	HB	69/69	66.4±8.5/69.1±8.4	PCR	7	43	19	1	48	20	0.000	8

*RFLP* restriction fragment length polymorphism, *PCR* polymerase chain reaction, *PB* population based, *HB* hospital based, *Con* control, *HWE* Hardy-Weinberg equilibrium, *NOS* Newcastle-Ottawa Scale

### Meta-analysis results

#### Analysis of heterogeneity

**Fig. 2:**
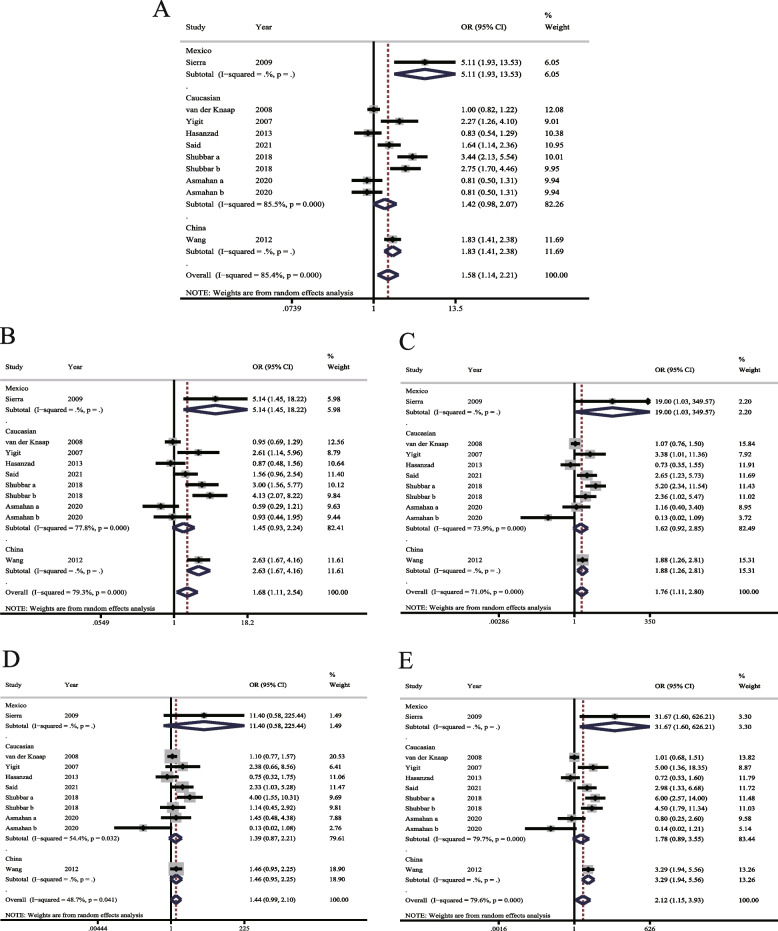
Forest plots of meta-analysis of ACE I/D polymorphism associated with susceptibility to prostate cancer. ACE I/D: angiotensin-converting enzyme insertion/deletion. **A** Model of allelic gene. **B** Model of recessive gene inheritance. **C** Model of dominant gene inheritance. **D** Model of heterozygous gene inheritance. **E** Model of homozygous gene inheritance

Significant heterogeneities across studies were identified in the models of allelic (*I*^2^ = 85.4%, *P* < 0.001), recessive (*I*^2^= 79.3%, *P* < 0.001), dominant (*I*^2^ = 71.0%, *P* < 0.001), heterozygous (*I*^2^= 48.7%, *P* = 0.041), and homozygous (*I*^2^= 79.6%, *P* < 0.001) gene, so the REMs were used (Fig. 2A–E).

#### Comparison of allele model

The results (Fig. 2A) showed that the allele D of the ACE I/D gene variant increased the risk of PCa compared with the allele I (D vs. I: OR= 1.58, 95% CI: 1.14–2.21, *P* = 0.007).

#### Comparison of other genetic models

According to analysis results, except for the heterozygous model (Fig. 2D), the differences in the models of recessive (Fig. 2B), dominant (Fig. 2C), and homozygous gene (Fig. 2E) reached significance (DD vs. DI + II: OR = 1.68, 95% CI: 1.11–2.54, *P* = 0.015; DD + DI vs. II: OR = 1.76, 95% CI: 1.11–2.80, *P* = 0.016; DI vs. II: OR = 1.44, 95% CI: 0.99–2.10, *P* = 0.055; DD vs.II: OR = 2.12, 95% CI: 1.15–3.93, *P* = 0.016).

#### Subgroup analysis

As shown in Fig. 2A, the heterogeneity in the allele model showed no marked decrease based on ethnicity. The subgroup analysis of seven studies with the controls meeting the HWE suggests a significant difference (OR= 1.70, 95% CI:1.18–2.46, *P*= 0.005), but the heterogeneity still had no significant reduction (*I*^2^= 84.2%, *P* < 0.001) (Supplementary Fig. 1A).

The heterogeneities across studies showed no marked decrease under the recessive, dominant, heterozygous, and homozygous gene models based on ethnicity (Fig. 2B–E). The subgroup analysis based on the controls meeting the HWE (Supplementary Fig. 1B–E) indicated that the differences of the pooled ORs were statistically significant in the dominant, recessive and homozygous gene models (DD + DI vs. II: OR = 1.84, 95% CI: 1.22–2.78, *P* = 0.004; DD vs. DI + II: OR = 1.86, 95% CI: 1.10–3.14, *P* = 0.021; DD vs.II: OR = 2.56, 95% CI: 1.32–4.96, *P* = 0.005), but heterogeneity did not decrease significantly. However, in the heterozygous gene model, heterogeneity reduced significantly (*I*^2^= 0.0%, *P* > 0.1), with the difference of the pooled OR significant (DI vs.II: OR= 1.35, 95% CI: 1.07–1.72, *P* = 0.013). Collectively, these data revealed an association of ACE I/D polymorphism with susceptibility to PCa.

### Detection of publication bias

The P-values of Egger’s test in the allelic, recessive, dominant, heterozygous, and homozygous models were 0.088, 0.115, 0.086, 0.105, and 0.077, respectively, which were all not statistically significant. Moreover, no obvious asymmetry was found in the funnel plots (Supplementary Fig. 2A–E). These results suggested no publication bias in our study.

### Sensitivity analysis

**Fig. 3:**
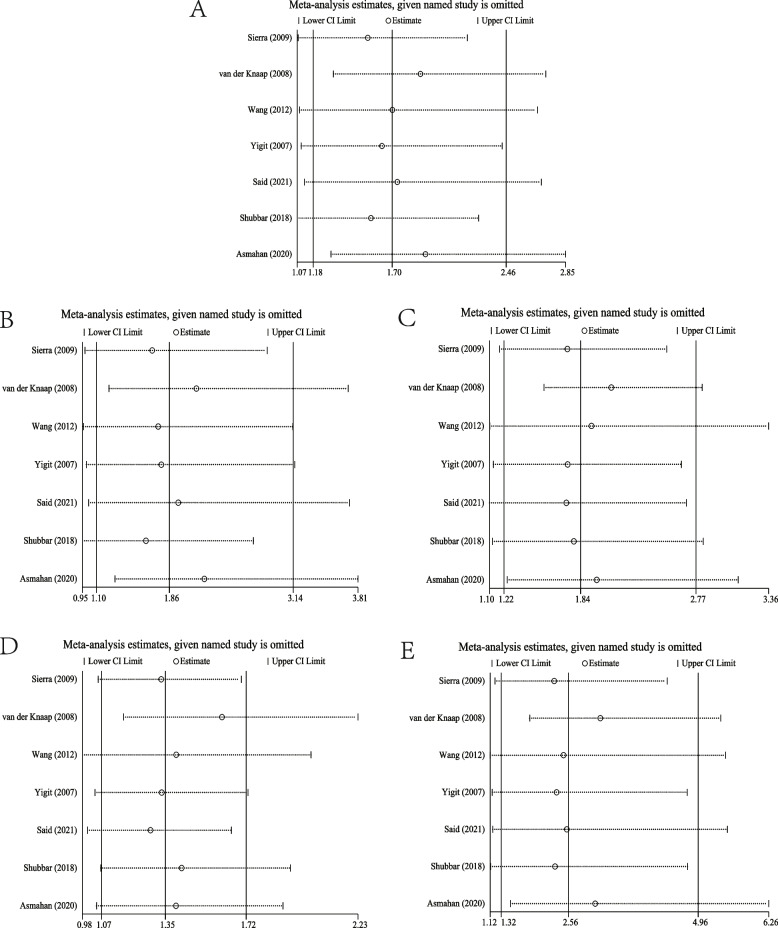
Sensitivity analysis of subgroup with genotypic frequencies satisfying HWE in the control group. **A** Model of allelic gene. **B** Model of recessive gene inheritance. **C** Model of dominant gene inheritance. **D** Model of heterozygous gene inheritance. **E** Model of homozygous gene inheritance. HWE, Hardy-Weinberg equilibrium

We performed sensitivity analysis by removing the relevant individual studies one by one for those which met the HWE in the control group (Fig. 3A–E). The results demonstrated no significant changes in the pooled ORs in the allelic (Fig. 3A), dominant (Fig. 3C), and homozygous genetic models (Fig. 3E). However, in the recessive and heterozygous models, the pooled ORs were significantly changed after the removal of four studies [10, 16, 18, 19] and one study [19], respectively. Therefore, the conclusions for the recessive and heterozygous models were somewhat unstable.

## Discussion

The objective of this meta-analysis aims to investigate the relationship between ACE I/D gene polymorphism and susceptibility to PCa. In accordance with strict inclusion and exclusion criteria, eight articles (10 studies) [10–12, 16–20] were identified in this systematic review. The analysis results determined an association of ACE I/D polymorphism with susceptibility to PCa, especially in the models of the allelic, dominant, recessive, and homozygous genes. At the same time, we noted that there was large heterogeneity in the analysis of these gene models (Fig. 2A–E). Heterogeneity did not reduce based on subgroup analysis of ethnicity, indicating ethnicity might not be a major source of heterogeneity. Of the eight articles included, six were the Caucasian population [10–12, 16, 17, 20], one Mexican [18], and one Chinese [19]. Therefore, although the differences of the Mexican and Chinese populations were statistically significant in the allelic, dominant, recessive, and homozygous models, we could not draw a conclusion yet because of the limited studies. Although the Caucasian population, which accounted for a large proportion, had no significant difference in all models, the conclusions could not be drawn for the time being due to the large heterogeneity. Therefore, the differences between ACE I/D polymorphism and prostate cancer susceptibility are inconclusive among the populations in different regions, which need to be verified by more studies in the future.

Furthermore, in the subgroup analysis based on genotype frequency in the control group meeting HWE (Supplementary Fig. 1A), the difference in the pooled OR was statistically significant in allelic models, but no significant decrease in heterogeneity. In addition, heterogeneity had no marked reduction in dominant, recessive, and homozygous gene models across studies with controls in HWE (Supplementary Fig. 1C, B, E). However, in the heterozygous model (Supplementary Fig. 1D), heterogeneity decreased significantly, and the difference in the pooled ORs was also statistically significant. Furthermore, Egger’s test showed no significant differences in all models, and the funnel plots did not show significant asymmetry (Supplementary Fig. 2A-E), suggesting no marked publication bias in this review. In the sensitivity analysis (Fig. 3), the conclusions in the dominant, allelic, and homozygous models were robust, while the findings in the recessive and heterozygous models were somewhat unstable. Collectively, this meta-analysis depicts an association of ACE I/D polymorphism with susceptibility to PCa.

A systematic review by Wang et al. [9], including five articles, reported that the OR was 1.56 (95% CI: 1.00–2.46) in the allele model, while no significant difference existed in the recessive gene model. However, the results in the allele model seemed to be not stable enough, for the 95% lower limit of the OR value was almost lower than 1. The sensitivity analysis in our study verified the robustness of the conclusions in the allelic model. Additionally, different from the findings in the study by Wang et al. [9], the result of this meta-analysis was significant in the recessive gene model. The difference in conclusions might be due to the fact that more studies were enrolled in our meta-analysis, thus making our meta-analysis more robust.

It is well-known that genetic variants may alter the function of proteins and individual susceptibility to cancer [10, 11]. The ACE gene is involved in the pathogenesis of cancer [9, 21]. Some polymorphic loci in the ACE gene may contribute to PCa development. Song et al.’s study [22] revealed that the use of ACE inhibitors or angiotensin receptor blockers in cancer patients reduced cancer recurrence and mortality by 40–25%. However, Siltari et al. reported that the use of ACE inhibitors could increase the risk of prostate cancer [23].

Limitations unavoidably exist in this study. First of all, only eight articles were identified in this study, with a sample size small, leading to some impact on the robustness of the findings. Then, only researches published in English were enrolled in the meta-analysis, and high-quality studies published in other languages or those not yet published may have been missed, causing potential publication bias. Third, the conclusions of the recessive and heterozygous models were somewhat unstable in the sensitivity analysis. Fourth, only subgroup analyses based on ethnicity and HWE were conducted to investigate the sources of heterogeneity across the studies, but other factors affecting heterogeneity failed to be determined. Fifth, due to the limited information provided by the included literature, no further exploration and analysis were carried out on the interrelationship between genes and genes, and between genes and environment.

In conclusion, this study indicates an association of ACE I/D gene polymorphism with susceptibility to PCa through the method of meta-analysis. Specifically, allele D, genotype DD+DI, and DD at the ACE I/D locus increase the susceptibility to PCa. Therefore, ACE I/D polymorphism can be adopted as a diagnostic and screening biomarker for PCa patients. Moreover, a certain reference for individualized immunotherapy for PCa patients according to ACE I/D polymorphism was offered in this meta-analysis. However, considering the limitations of this study, more rigorously designed and large-scale researches are still needed to further verify the relationship between ACE I/D polymorphism and the PCa risk.

## Supplementary Information

**Additional file 1: Supplementary Table 1.** Retrieval steps and results in PubMed (The retrieval time: From inception to June 1, 2022).

**Additional file 2: Supplementary Figure 1.** Forest plots of ACE I/D polymorphism associated with susceptibility to prostate cancer based on HWE. ACE I/D: angiotensin-converting enzyme insertion/deletion. A: Model of allelic gene; B: Model of recessive gene inheritance; C: Model of dominant gene inheritance; D: Model of heterozygous gene inheritance; E: Model of homozygous gene inheritance. HWE: Hardy-Weinberg equilibrium.

**Additional file 3: Supplementary Figure 2.** Funnel plot to detect potential publication bias. A: Model of allelic gene; B: Model of recessive gene inheritance; C: Model of dominant gene inheritance; D: Model of heterozygous gene inheritance; E: Model of homozygous gene inheritance.

## Data Availability

The datasets adopted during the meta-analysis are available on reasonable request from the corresponding author.

## Abbreviations

ACE I/D: Angiotensin-converting enzyme insertion/deletion
OR: Odds ratio
PCa: Prostate cancer
RAAS: Renin angiotensin aldosterone system
NOS: Newcastle-Ottawa Scale
CI: Confidence interval
REM: Random-effects model
FEM: Fixed-effects model
HWE: Hardy-Weinberg equilibrium
