# Supplementary material for: Association of angiotensin-converting enzyme insertion/deletion (ACE I/D) gene polymorphism with susceptibility to prostate cancer: an updated meta-analysis
Source: World J Surg Oncol. 2022 Nov 4;20:354. doi: 10.1186/s12957-022-02812-x (PMC9635097; doi:10.1186/s12957-022-02812-x)
Supplement: Supplementary file 1 — Additional file 1: Supplementary Table 1. Retrieval steps and results in PubMed (The retrieval time: From inception to June 1, 2022). [file 12957_2022_2812_MOESM1_ESM.docx]

**Supplementary Table 1.** Retrieval steps and results in PubMed (The retrieval time: From inception to June 1, 2022).

| Search | Query | Items found |
| --- | --- | --- |
| #1 | Prostate cancer[MeSH terms] | 143024 |
| #2 | "Prostate cancer" [Title/Abstract] OR "prostate carcinoma"[Title/Abstract] OR “prostate tumor”[All fields] | 137017 |
| #3 | #1 OR #2 | 177094 |
| #4 | Angiotensin-converting enzyme [MeSH terms] | 14486 |
| #5 | “Angiotensin-converting enzyme” [Title/Abstract] OR "ACE" [Title/Abstract] | 66204 |
| #6 | #4 OR #5 | 68959 |
| #7 | Polymorphism [MeSH terms] | 296660 |
| #8 | “Polymorphism” [Title/Abstract] OR “single nucleotide mutation” [Title/Abstract] OR “mutation”[Title/Abstract] | 580028 |
| #9 | #7 OR #8 | 727119 |
| #10 | #3 AND #6 AND #9 | 15 |
