# Supplementary figures and images for: Association of angiotensin-converting enzyme insertion/deletion (ACE I/D) gene polymorphism with susceptibility to prostate cancer: an updated meta-analysis
Source: World J Surg Oncol. 2022 Nov 4;20:354. doi: 10.1186/s12957-022-02812-x (PMC9635097; doi:10.1186/s12957-022-02812-x)

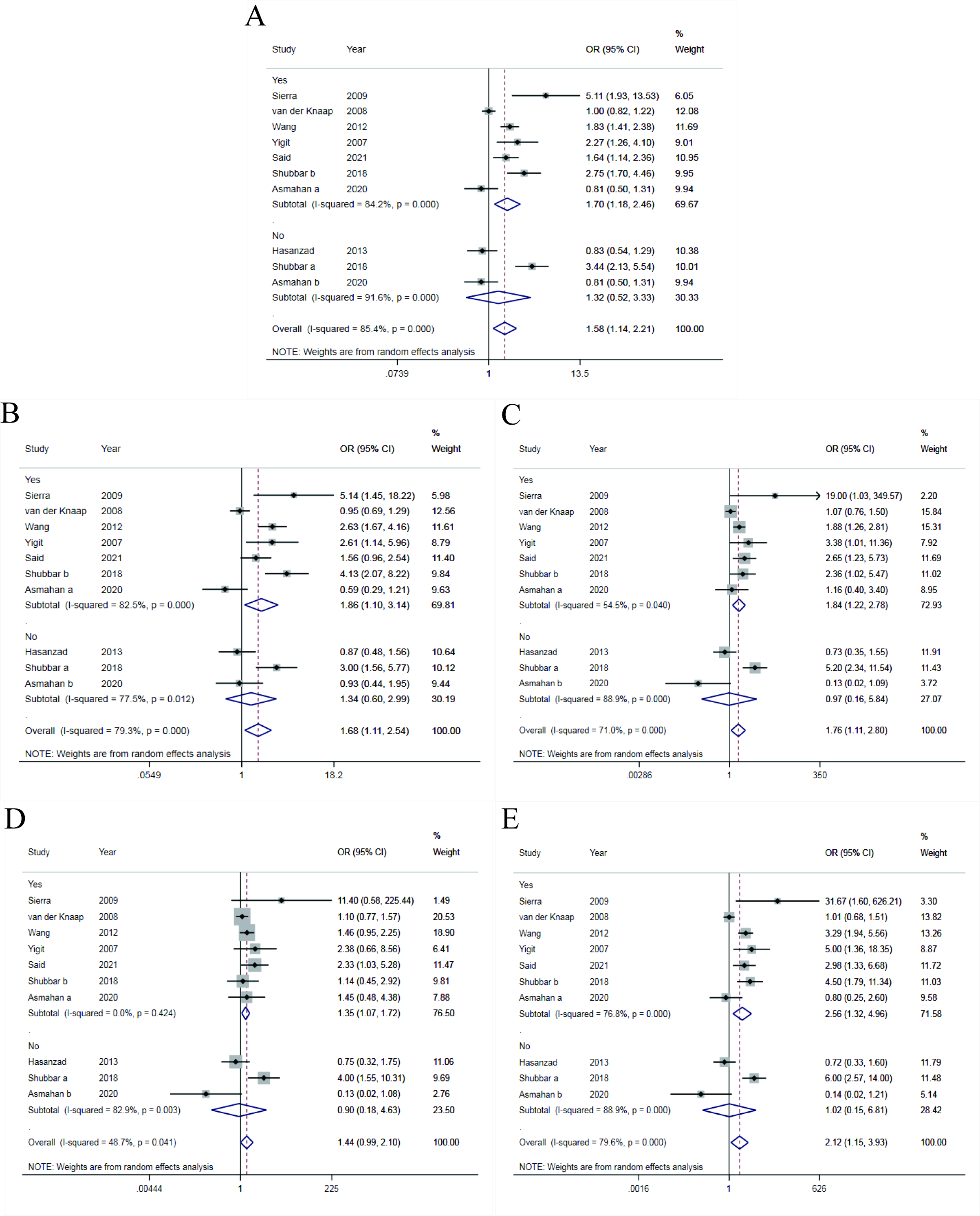

Supplement: Supplementary file 2 — Additional file 2: Supplementary Figure 1. Forest plots of ACE I/D polymorphism associated with susceptibility to prostate cancer based on HWE. ACE I/D: angiotensin-converting enzyme insertion/deletion. A: Model of allelic gene; B: Model of recessive gene inheritance; C: Model of dominant gene inheritance; D: Model of heterozygous gene inheritance; E: Model of homozygous gene inheritance. HWE: Hardy-Weinberg equilibrium. [file 12957_2022_2812_MOESM2_ESM.tif]

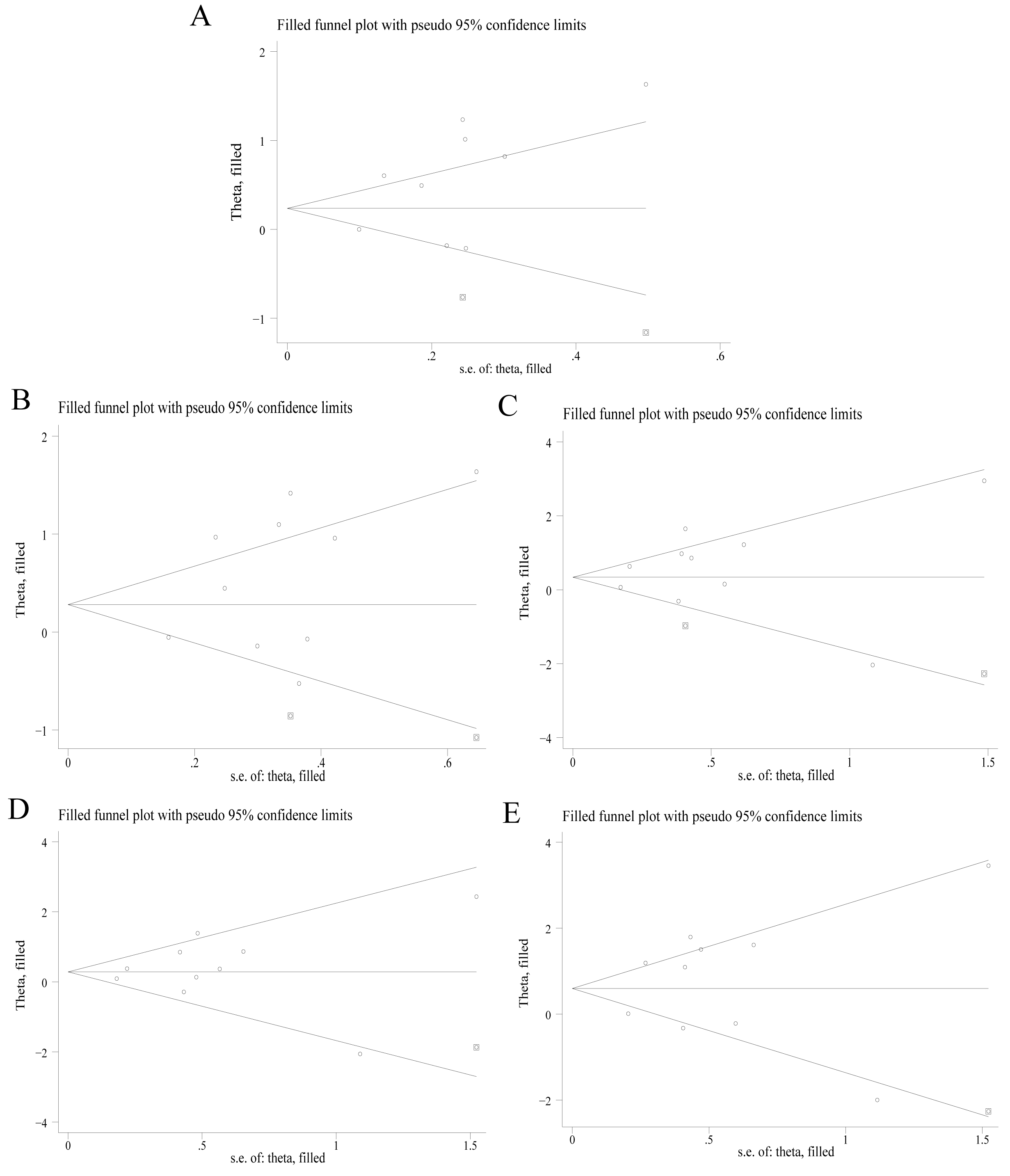

Supplement: Supplementary file 3 — Additional file 3: Supplementary Figure 2. Funnel plot to detect potential publication bias. A: Model of allelic gene; B: Model of recessive gene inheritance; C: Model of dominant gene inheritance; D: Model of heterozygous gene inheritance; E: Model of homozygous gene inheritance. [file 12957_2022_2812_MOESM3_ESM.tif]
